# Safety assessment of a proprietary preparation of a novel Probiotic, *Bacillus coagulans*, as a food ingredient

**DOI:** 10.1016/j.fct.2009.02.018

**Published:** 2009-06

**Authors:** J.R. Endres, A. Clewell, K.A. Jade, T. Farber, J. Hauswirth, A.G. Schauss

**Affiliations:** a AIBMR Life Sciences Inc., 4117 South Meridian, Puyallup WA 98373, USA; b ToxaChemica, International, 11430 Strand Drive, Rockville MD 20852, USA; c Van Gemert and Hauswirth, LLC, 3222 Green Forest Ct., Ellicott City MD 21042, USA

**Keywords:** A/G, albumin to globulin ratio, Alb, albumin, ALKP, alkaline phosphatase, ALT, alanine transaminase, ANOVA, analysis of variance, APTT, activated partial thromboplastin time, AST, aspartate transaminase, ATCC, American Type Tissue Collection, BC30 or GBI-30-SF, GanedenBC^30™^, Bw, body weight, CFU, colony forming unit, Creat, creatinine, DMSO, dimethyl sulfoxide, EEC, European Economic Community, EC, European Community, ELISA, enzyme-linked immunosorbent assay, EU, European Union, FBS, fetal bovine serum, GGT, gamma-glutamyltransferase, GRAS, generally recognized as safe, Hct, hematocrit, Hgb, hemoglobin, MC, methyl cellulose, MCH, mean corpuscular hemoglobin, MCHC, mean corpuscular hemoglobin concentration, MCV, mean corpuscular volume, MPV, mean platelet volume, NOAEL, no observed adverse effect level, OECD, Organization for Economic Cooperation and Development, Plt, platelet, PT, prothrombin time, RBC, red blood cell, RDW, red blood cell distribution width, Retic, reticulocyte, S.D., standard deviation, T Bili, total bilirubin, T chol, total cholesterol, T Prot, total protein, UV, ultraviolet, v/v, volume per volume, WBC, white blood cell, *Bacillus coagulans*, BC30, Functional foods, GRAS, Gut flora, Probiotics

## Abstract

It has been demonstrated that some strains of *Bacillus coagulans* can survive extremes of heat, acidity of the stomach, and bile acids, to which commonly consumed probiotics are susceptible. A toxicological safety assessment was performed on a proprietary preparation of *B*. *coagulans* – GanedenBC^30™^ – a novel probiotic. Seven toxicologic studies were conducted and included: *in vitro* bacterial reverse mutation assay; *in vitro* chromosomal aberration assay; micronucleus assay in mice; acute and 90 day subchronic repeated oral toxicity studies were conducted in Wistar Crl:(WI) BR rats; acute eye and skin irritation studies were conducted in rabbits.

The results of this toxicological safety assessment indicate that GanedenBC^30™^*B*. *coagulans* does not demonstrate mutagenic, clastogenic, or genotoxic effects. Furthermore, the results of the acute and 90-day subchronic oral toxicity studies in rats resulted in the conclusion of a NOAEL greater than 1000 mg/kg per day. Since the concentration of the cell mass used in the 90-day study was 1.36 × 10^11^ CFUs/g, this corresponds to 95.2 × 10^11^ CFUs for a 70 kg human and since the suggested human dose is in the range of 100 × 10^6^ to 3 × 10^9^ CFUs, this gives a safety factor ranging from 3173 to 95,200 times. Based upon scientific procedures and supported by history of use, GanedenBC^30™^ is considered safe for chronic human consumption.

## 1 Introduction

Probiotics are defined as viable organisms (generally bacteria or yeast) that have demonstrated beneficial effects on the health of a host (Lee, 1999). While the concept of utilizing probiotics for human health has been around for over 100 years (De Vecchi and Drago, 2006), recently a great deal of interest has been focused on the importance of probiotic bacteria in treating or preventing specific disorders such as irritable bowel syndrome, eczema, allergies, *Helicobacter pylori* infection and sequelae, as well as for support of intestinal and immunological health (Tappenden and Deutsch, 2007; Quigley, 2007; Rastall et al., 2005; Hyronimus et al., 2000; Spiller, 2008; McFarland and Dublin, 2008; Lesbros-Pantoflickova et al., 2007; Ouwehand, 2007).

Probiotic bacteria such as the *Lactobacillus* species are very sensitive to normal physiological conditions such as the very low pH of the stomach and bile salts when consumed. In addition, the viability of these bacteria is affected by manufacturing methods as well as storage and shipping conditions (Bezkorovainy, 2001; Ljungh and Wadstrom, 2006; Graff et al., 2008). On the contrary, some strains of *Bacillus coagulans* are able to survive the extremes of heat, acidity of the stomach and bile acids – although in general, the strains of this species are quite heterogeneous (Patel et al., 2006; De Clerck et al., 2004; De Vecchi and Drago, 2006; Hyronimus et al., 2000; Katsutoshi et al., 2003). Strains with these qualities have an increased chance of survival through the gastrointestinal tract, thus allowing for transient population of the small and large intestines by *B*. *coagulans* (Adami and Cavazzoni, 1999). *B*. *coagulans*, when taken orally, has also shown beneficial effects on the intestinal environment, stool frequency and characteristics, and dermal attributes in animals and humans (Adami and Cavazzoni, 1999; Donskey et al., 2001; Katsutoshi et al., 2003).

The majority of probiotics studied and sold in the market today are classified as lactic acid producing organisms (De Vecchi and Drago, 2006; Lee, 1999), including many *Lactobacillus* species. Until 1974 *B*. *coagulans* was classified as *Lactobacillus sporogenes.* Bergey’s Manual (Buchanan and Gibbons, 1974; Bergey’s Manual, 1974) reclassified this bacterium as *B*. *coagulans* because, although it shares taxonomic characteristics with the other *Lactobacillus* species such as producing lactic acid, none of the latter are spore-forming (Sanders et al., 2001; Gandhi, 1994). As 16S RNA ribotyping, in addition to fatty acid analysis, became available and routine for identification and classification of various bacteria, it has become clear that *B*. *coagulans* is the correct and well-accepted taxonomic classification.

The reported history of *B*. *coagulans* dates back to 1915, when an outbreak of coagulation in evaporated milk was described in the Iowa Agricultural Experiment Station (Sarles and Hammer, 1932). In 1932, *L*. *sporogenes* was first isolated and described by Horowitz-Wlassowa and Nowotelnow (Gandhi, 1994). The observers noted that the milk was curdled, slightly bitter, and with flavor of a cheesy nature that was not at all unpleasant.

More recently, in 1978 a patent was issued for a method (USPTO No. 4,110,477) for improving the flavor and shelflife of natto (a traditional Japanese food made from fermented soybeans) by the addition of *B*. *coagulans* along with its close relative *Bacillus natto (Bacillus subtilis)*, which conventionally has been used to manufacture this commonly consumed food.

While many strains of *B*. *coagulans* have been widely consumed around the world for decades at the least, to the best of the authors’ knowledge no comprehensive toxicologic assessment for *B*. *coagulans* has been published in the public domain. Unlike other non-pathogenic species of bacteria, *B*. *coagulans* is not generally considered a natural part of the gastrointestinal tract flora. Because it lacks the ability to adhere to the intestinal epithelium, it is completely eliminated in four to five days unless chronic administration is maintained (Donskey et al., 2001). Therefore, we have conducted a thorough assessment to evaluate the safety of chronic oral consumption of GanedenBC^30™^. The purpose of this constellation of research is to present the safety assessment of a proprietary strain of *B*. *coagulans* known as GanedenBC^30™^ and includes the following *in vitro* and *in vivo* toxicology studies: Ames mutagenicity, chromosomal aberration, mouse micronucleus test, acute eye irritation and acute skin irritation in the rabbit, acute oral toxicity in the rat, as well as a 90-day subchronic oral toxicity in the rat.

## 2 Materials and methods

### 2.1 Test product

The test product, GanedenBC^30™^, supplied by Ganeden Biotech, Inc. (Mayfield Heights, OH, USA) is sold as a dietary ingredient for use in functional foods and dietary supplements. The organism is a gram-positive spore-forming rod that is aerobic to microaerophilic in nature. Its size is 0.9 μm × 3.0 μm × 5.0 μm. GanedenBC^30™^ is manufactured as a pure cell mass consisting solely of *B*. *coagulans*. The pure cell mass is spray-dried with maltodextrin to achieve the desired concentration of 15 × 10^9^ CFU/g for the finished product. For the purpose of these toxicologic studies, pure, uncut GanedenBC^30™^ was used. The concentration varies slightly from batch to batch and therefore is reported for each study.

### 2.2 Bacterial reverse mutation (Ames) study

Five strains of bacteria, including four strains of *S*. *typhimurium* (TA98, TA100, TA1535, TA1537) and one strain of *E. coli* (WP2 [uvrA]) were obtained from Xenometrix GmbH (Allschwil, Switzerland). This study is well known and has been previously described (Ames et al., 1975; Maron and Ames, 1983). The test article, obtained at a concentration of 4.5 × 10^10^ CFU/g, was mixed with sterile water not more than 30 min prior to the start of the experiment. The study was performed both with and without an S9 activation system. The Aroclor™ 1254-induced rat liver S9 was purchased from Trinova Biochem GmbH (Giessen, Germany). A cytotoxicity assessment was performed on test doses of 5–5000 μg/plate to determine the appropriate dose range for the assay. Doses used in the study were: 10, 50, 100, 500, and 5000 μg/plate. Experiments were performed in triplicate.

Positive controls in the experiment without S9 activation included 2-nitrofluorene (CAS#607-57-8) for strain TA98, sodium-azide (CAS#26628-22-8) for strains TA100 and TA1535, 9-aminoacridine (CAS#52417-22-8) for strain TA1537 and methyl-methansulfonate (CAS#66-27-3) for strain WP2. Positive controls in the experiment with S9 activation included benzo(a)pyrene (CAS#50-32-8) for strains TA98 and TA1537, and 2-aminoanthracene (CAS#613-13-8) for strains TA100, TA1535 and WP2. All strains were tested for spontaneous revertant colonies using distilled water as a negative control.

Frozen stock cultures were grown overnight at 37 ± 2 °C to a density of 10^9^ cells/ml. Sterile culture tubes were filled with 0.1 ml of test article culture or control, 0.5 ml of S9/cofactor mix or 0.5 ml phosphate buffer (pH 7.4), and 2.0 ml top agar. The mixture was spread onto minimal glucose plates and incubated at 37 °C ± 2 °C for 72 ± 4 h. The number of revertant colonies per plate was determined by hand counting. Criteria for a valid assay included sensitivity of TA98, TA100, TA1535, TA1537 and WP2 to UV light, sensitivity of *S. typhimurium* strains to crystal violet, resistance of strains TA98 and TA100 to ampicillin, reversion rates within historical ranges, and a threefold increase in revertant colonies with exposure to positive controls. A response was considered positive if there was over three times the background average number of revertant colonies on a plate (two times for strain TA100), or if there was a dose-related increase in colonies. If these criteria were not met, the test article was considered non-mutagenic.

Statistical regression analysis was not necessary based on the results. Because the definitive assay yielded negative results, an independent repeat assay was performed, with an increase in S9 in the S9/cofactor mix from 4% to 10%. The experiments were conducted according to Organization for Economic Cooperation and Development (OECD) Guidelines for the Testing of Chemicals: Bacterial Reverse Mutation Test (Guideline 471, as adopted July 21, 1997).

### 2.3 Micronucleus assay in mice

Male mice of strain BALB/dByJNarl, aged 7–8 weeks were obtained from the National Laboratory Animal Center (Taipei, Taiwan). Body weights at the start of the study were 21.9–25.9 grams. Animals assigned randomly to five groups of five, and were housed with five animals per cage. Conditions included 50 ± 20% relative humidity, 21 ± 2 °C temperature, and a 12-h light–dark cycle. Animals had free access to Laboratory Autoclavable Rodent Diet 5010 (PMI^®^ Nutrition International, Inc., MO, USA). Drinking water was available *ad libitum*.

Animals were weighed and observed for signs of illness or other abnormalities at the start of the study. Randomization was generated with Microsoft Excel, 2003 SP2. Water for injection was utilized as the negative control, and was used as the vehicle to prepare the test article to desired concentration on the same day as administration. The test article was obtained at a concentration of 1.93 × 10^11^ CFUs/g. Mitomycin C (Taiwan Biotech Co. Ltd., Taiwan) served as the positive control. Doses were administered by oral route, except for the positive and negative controls which were administered only once by intraperitoneal injection on the last test article administration day.

Doses of test article included 500, 1000 and 2000 mg/kg bw/day given for three days. Body weights were measured on days 1–5. Blood samples were obtained by tail trimming approximately 48 h after the last administration. Samples were smeared on acridine orange-coated slides, and 2000 reticulocytes were scored under a fluorescence microscope for the presence of micronuclei and the slides were blind coded. The proportion of reticulocytes to total erythrocytes was an indicator to evaluate bone marrow toxicity. Percentages were determined by flow cytometry and were based on analysis of 50,000 erythrocytes.

Criteria for a valid test included negative control data comparable to historical control data, and positive control data of significantly increased levels of micronucleated reticulocytes compared to the negative control (*p* ⩽ 0.05, *t*-test). Data was analyzed using one-way analysis of variance (ANOVA) (SigmaStat, V3.11, 2004). A *p* value of ⩽0.05 was used as criterion for statistical significance. If statistically significant, the data were analyzed for a dose–response relationship. A repetition of the experiment was performed if positive findings were not dose dependant.

### 2.4 *In vitro* chromosomal aberration assay

Chinese hamster ovary cells (CHO-K1) were obtained from American Type Culture Collection (ATCC, Rockville, MD, USA) as repository number CCL-61. The cell line was tested by ELISA (Böhringer, Mannheim) and found to be free of mycoplasma contamination. Cells used in the assay were within five passages from the frozen stock to assure karyotypic stability.

Cells were maintained as monolayers in McCoy’s 5A medium (Sigma, USA) supplemented with 10% fetal bovine serum (FBS), 0.22% sodium bicarbonate, 2 mM l-glutamine, and 1% penicillin–streptomycin solution, at 7.0–7.2 pH in a humidified incubator at 37 °C and 5% CO_2_ in air. Post-mitochondrial fraction (S9) of the liver homogenate from Sprague-Dawley rats induced with Aroclor 1254 (Moltox, Inc. USA) was used for metabolic activation. The final concentration of S9 was 1% (v/v).

A dose range finding test was done according to OECD guidelines, which suggests the greatest concentration of the test article tested should produce greater than 50% cytotoxicity, if possible, with a maximum concentration of 5000 μg/ml for relatively non-cytotoxic substances. The test article was obtained at a concentration of 1.93 × 10^11^ CFUs/g and was prepared at 5 mg/ml in medium and was further diluted with medium to the final concentration desired. Five concentrations were utilized in the chromosomal aberration assay, including 312.5, 625, 1250, 2500 and 5000 μg/ml. One micromolar mitomycin C (Roche, USA) served as the positive control in the schemes without S9 activation, while 40 μM cyclophosphamide (Sigma, USA) served as the positive control in schemes with S9 activation. Both positive controls were prepared in DMSO, with a final concentration of 0.5% in culture medium. Culture medium alone served as the negative control.

Three treatment schemes were utilized, including incubation of the test article with cells for 3 h both with and without an S9 metabolic activation system (Schemes I and II, respectively), and incubation for 20 h without S9 (Scheme III). Due to only two analyzable concentrations in treatment scheme III (312.5 and 625 μg/ml), this scheme was repeated with 78.125, 156.25, 312.5 and 1250 μg/ml concentrations. Colcemid was added to all cell culture medium to a final concentration of 0.1 μg/ml for the final 2 h of incubation prior to harvesting cells.

Cells were evaluated for cytotoxicity and chromosomal integrity by cell counting on fixed slides that were blind coded. Two cultures were scored for each dose, and 100 metaphases were observed for each culture. Chromosomal and chromatid aberrations were scored, and percents of structurally aberrant cells were calculated, excluding cells with chromosome or chromatid gaps.

If more than 3% aberrant cells were observed, the percent was analyzed by a one-tail binomial test and compared pair-wisely to the negative control. If a concentration gave a significant result, a trend test was performed to determine the existence and extent of dose–responsiveness.

### 2.5 Acute oral toxicity study in rats

Wistar Crl:(WI) BR rats were obtained from Toxi-Coop Kkt. (Budapest, Hungary). Rats were housed five per cage at a temperature of 22 ± 3 °C, a relative humidity of 30–70%, and a 12 h light–dark cycle.

Animals received Ssniff^®^ SM R/M-Z+H complete diet for rats, produced by Ssniff Spezialdiäten GmbH (Soest, Germany). Tap water was available *ad libitum*. Randomization and statistical analysis was performed with SPSS PC+ software (SPSS Inc., Chicago, IL, USA). Body weights at randomization were 190–200 g for males, and 171–180 g for females. Each group consisted of 5 male and 5 female rats.

Detailed clinical observations were made prior to exposure of the animals to the test article. The test article was obtained at a concentration of 1.04 × 10^11^ CFUs/g, and was diluted in 1% methylcellulose (Dow Chemical TEVA Gyógyszergyár ZRt., Hungary) 30 min prior to administration. A single oral dose of 5000 mg/kg/bw of test article was administered to the treatment group and MC solution of 1% was administered to the control group by oral gavage. Following administration, the animals were observed for clinical signs for four continuous hours, and then twice daily (once daily on weekends) for 14 days.

Observations were focused on skin, fur, eyes, mucous membranes, autonomic activity, circulatory and central nervous system, somatomotor activity, behavior pattern, tremors, convulsions, salivation, stool consistency, lethargy, sleep, changes in gait, posture and response to handling. Animals were weighed on the day of treatment, and on days 2, 8, and 15. A gross pathological organ examination (and histopathological examination if necessary) was performed after the animals were sacrificed with diethyl ether (Reanal, Budapest, Hungary) on the 15th day.

The study was performed according to the United States FDA Toxicological Principles for the Safety Assessment of Direct Food Additives and Color Additives Used in Food Redbook II Draft Guidance, Acute Oral Toxicity Tests (1993), as well as the OECD Guideline for the Testing of Chemicals No. 423; Acute Oral Toxicity – Acute Toxic Class Method, adopted December 17, 2001.

### 2.6 Subchronic 13-week oral toxicity study in rats

Wistar Crl:(WI) BR rats were obtained from Toxi-Coop Kkt. (Budapest, Hungary). The animals were housed five per cage, at a temperature of 22 ± 3 °C, a relative humidity of 30–70%, and a 12-h light–dark cycle.

Animals received Ssniff^®^ SM R/M-Z+H complete diet for rats (Ssniff Spezialdiäten GmbH, Soest, Germany). Tap water was available *ad libitum*. The drinking water was analyzed once after the study and was determined to be free of contaminants. An equal number of animals from each weight and sex group were randomized to the two experimental groups. Body weights of the animals at randomization were 192–218 g for males, and 159–181 g for females. Each group consisted of 20 rats as 10 males and 10 females.

The test article was obtained at a concentration of 1.36 × 10^11^ CFUs/g, and weighed daily and suspended in 1% methylcellulose (Dow Chemical, TEVA Gyógyszergyár ZRt., Hungary) in distilled water. Concentration and homogeneity of five parallel samples of the test suspensions and controls were checked by gravimetry during weeks 1, 4, 8 and the last week. Doses of 0, 100, 300 and 1000 mg/kg bw/day in a volume of 10 ml/kg was given daily by oral gavage for 90 (males) or 91 (females) days. Weekly adjustments were made for body weight changes.

Clinical observations were made once daily following treatment at approximately the same time each day. Detailed clinical observations were made on all animals outside the cage using a standard method both before the first exposure, and once a week thereafter. The focus of the observations was as described in the acute oral toxicity study above. All animals were weighed on the day of randomization, on day one, once weekly thereafter, and on the day of autopsy. Food was weighed weekly, and the average food consumption per animal was calculated. Water consumption was measured over a 24-h period weekly, and average water consumption per animal was calculated.

On the 13th week of administration, sensory reactivity to different types of stimuli (auditory, visual and proprioceptive) was measured, as well as general physical condition and behavior of animals. A modified Irwin test was performed (Irwin, 1968). Hematology and clinical chemistry results were evaluated at the termination of the study. Animals were fasted for 16 h prior to blood collection. Animals were sacrificed with diethyl ether (Reanal, Budapest, Telepes Street 53, Hungary), and blood samples were collected by heart puncture.

Gross pathological examination was performed on all organs after sacrificing the animals, including organ weight and appearance. A histopathological examination was performed on the control group and the highest dose group, and on the middle dose groups when appropriate.

Statistical analysis was performed for body weights, food consumption, hematology, clinical chemistry and organ weights. Randomization and statistical analysis were performed with SPSS PC+ software (SPSS Inc., Chicago, IL USA). Barlett’s homogeneity of variance between groups was determined, and where there was no significant heterogeneity detected, a one-way ANOVA was performed. If statistically significant results were obtained, Dunnett’s test was used to assess the inter-group differences. If positive results were detected, inter-group comparisons were performed using Mann-Whitney *U*-test.

The study was performed according to the United States FDA Toxicological Principles for the Safety Assessment of Food Ingredients Redbook 2000, IV.C.3a; Short-term Toxicity Studies with Rodents (2003), as well as the OECD Guideline for the Testing of Chemicals No. 408; Repeated Dose 90-Day Oral Toxicity Study in Rodents, adopted September 21, 1998.

### 2.7 Acute eye irritation study in rabbits

Male New Zealand White rabbits (age 11 weeks) were obtained from Tetrabbit Kft. (2173 Kartal, Császár út 135, Hungary). Both eyes of animals were examined 24 h prior to the start of the study. Animals showing eye irritation, ocular defects or pre-existing corneal injury were not used in the experiment. The animals weighed 2521–3000 g at the beginning of the study, and 2780–3143 g at the end of the study. Animals were housed individually in metal cages at 20 ± 3 °C, relative humidity of 30–70% and a 12 h light–dark cycle. Animals were fed PURINA Base-Lap gr. diet for rabbits *ad libitum* from Agribands Europe, (H-5300 Karcag, Madarasi road, Hungary). Tap water was routinely analyzed for contaminants and was available *ad libitum*.

Test article was used at a concentration of 1.93 × 10^11^ CFU/g. The dosage for the study was 0.1 g of the undiluted cell mass. Three healthy male animals were selected, and the dose was placed into the conjunctival sac of the left eye of a single rabbit. After determination that the pain reaction was very low and that anesthesia was not required with application of the test article, the dose was added to the eyes of the remaining two animals, with the untreated right eyes serving as the control. Eyes were not washed after the application.

Eyes were examined at 1, 24, 48 and 72 h after the treatment for severity, nature and duration of reactions. The duration of the observation period was sufficient to determine the reversibility or irreversibility of any changes. Any clinical signs of toxicity or signs of ill-health of the animals were recorded during the study. Eye irritation scores were evaluated according to the Draize (1977) and OECD 405 (April 24, 2002) scoring systems. The study was performed in accordance with OECD Guidelines for Testing of Chemicals No. 405; Acute Eye Irritation/Corrosion, adopted April 24, 2002.

### 2.8 Acute skin irritation study in rabbits

Male New Zealand White rabbits (age 11 weeks) were obtained from Tetrabbit Kft. (2173 Kartal, Császár út 135, Hungary). Housing, feeding and drinking were the same as described above for the eye irritation study. Animals weighed 2525–2624 g at the beginning of the study, and 2712–2834 g at the end of the study.

Test article was used at a concentration of 1.93 × 10^11^ CFUs/g. An undiluted dose of 0.5 g was moistened sufficiently with water to ensure good contact with the skin, and a patch test was applied to approximately 6 cm^2^ of intact skin on three animals, starting with just one animal to ensure no corrosive or severe irritation. Sterile gauze pads and adhesive hypoallergenic plaster kept the test article in place. The trunks of the animals were wrapped in plastic wrap for 4 h, which was the duration of the test item exposure. After 4 h, the test item was removed by washing the animals with body temperature water. Untreated skin on each animal served as the negative control.

Animals were examined for erythema and edema at 1, 24, 48 and 72 h after the test article removal. The test article was evaluated according to the Draize (1959) method (OECD 404, 2002) for any irritant effect. The study was performed in accordance with OECD Guidelines for Testing of Chemicals No. 404; Acute Dermal Irritation/Corrosion, adopted April 24, 2002.

## 3 Results

### 3.1 Bacterial reverse mutation (Ames) study

The bacterial reverse mutation assay was performed to evaluate whether Ganeden *B. coagulans* cell mass has mutagenic properties. All criteria for a valid bacterial reverse mutation (Ames) study as described in the materials and methods section were met. The utility of the AMES study for a biological test article such as *B. coagulans*, may be questionable. The study was performed both as a typical part of a toxicologic safety assessment for a natural product as well as to investigate for any mutagenic properties that the bacteria or its fermentate may have. While the negative results of the study were encouraging, when considered alone the data may be less meaningful and therefore additional studies such as the chromosome aberration assay and mouse micronucleus study were employed to further investigate genotoxicity and clastogenicity.

There were no revertants exceeding three times the background average either with or without the S9 metabolic activation system. In addition, no dose-dependent increase in revertants was observed. In conclusion, the results of this study showed that the *B. coagulans* cell mass, GanedenBC^30™^ had no mutagenic effect for any strain used in this test. Furthermore, the results of the repeat assay confirmed the results of the definitive assay.

### 3.2 Micronucleus assay in mice

The micronucleus test was conducted to investigate for the formation of micronuclei containing chromosome fragments or whole chromosomes, which are indicative of cytogenetic damage. There were no differences in body weight between the treatment groups compared to the control group and no signs of toxicity were noted in clinical observations following administration of the test article at doses of 500, 1000 and 2000 mg/kg bw/day. Animals in the positive control group showed a significant increase in the frequency of micronuclei compared to the negative controls. None of the treatment groups were positive for statistically significant induction of micronuclei in reticulocytes, and the ratio of reticulocytes to total erythrocytes in these groups showed no significant decrease compared to the negative control group. The average reticulocyte to total erythrocytes ratio in the negative control group was 3.87%. The treatment groups were 3.69%, 3.65% and 3.69% in the 500, 1000 and 2000 mg/kg bw/day groups respectively. The positive control caused a 32.8% decrease in the ratio. This study indicates that Ganeden BC^30™^ did not cause signs of toxicity in the bone marrow of the mice in the range of the doses tested.

The incidence of micronucleated reticulocytes in the peripheral blood per 1000 reticulocytes was 1.8 ± 0.8 in the negative control group, which was within the historical reference range. The positive control group had a mean frequency of 31.2 ± 5.5, which was a statistically significant increase compared with the negative control group. The test article dose groups had 1.3 ± 1.2, 2.2 ± 1.0 and 0.9 ± 0.4 micronucleated reticulocytes per 1000 reticulocytes, at the test dose levels of 500, 1000, and 2000 mg/kg bw/d respectively. These values were not statistically significant, and thus did not demonstrate any signs of toxicity with administration of Ganeden BC^30™^ in the mouse peripheral blood micronucleus assay.

### 3.3 *In vitro* chromosomal aberration assay

The purpose of performing the chromosome aberration assay in cultured mammalian cells is to investigate for any potential the test article may have for causing structural damage to either chromosomes or chromatids.

Cells in the negative control group had 20 ± 2 chromosomes upon karyotypic analysis. The percentage of chromosomal aberrations measured in the negative control groups was zero. None of the dose levels of Ganeden BC^30™^ tested produced any statistically significant increase in aberrant cells, while the positive control groups did induce a significant increase when compared with the negative controls as expected. Therefore, under the conditions of the assay, Ganeden BC^30™^ produced a negative response for induction of structural chromosomal aberrations both with and without the metabolic activation system in Chinese hamster ovary cells.

### 3.4 Acute oral toxicity study in rats

A 14-day oral toxicity study in rats was performed to investigate the test article for acute toxicity. The results of such studies may provide preliminary toxicity data that is useful in determining appropriate dose levels for future repeated-dose oral toxicity studies as well as determining possible target organs that should be closely examined in such toxicity studies of a longer duration.

A single oral dose of 5000 mg/kg bw produced no treatment-related signs in any of the animals. Neither weight-loss nor changes in body weight resulted with the treatment compared with the control group. All of the organs examined in both the male and female dose groups were free from any gross pathological changes and thus, per OECD Guideline No. 423, histopathological examination was not performed. There was no evidence of any toxicity in the acute toxicity study as the results were unremarkable.

### 3.5 Subchronic 13-week oral toxicity study in rats

A 13-week repeated-dose toxicity study was performed in rats to determine a NOAEL for defined toxicological endpoints and is used to establish a safe chronic oral dose for humans. Ganeden BC^30™^ was administered orally by gavage at doses of 100, 300 and 1000 mg/kg bw/day for 90 consecutive days. There were no deaths and no treatment-related signs were observed throughout the 13-week treatment period in any of the groups. Appearance and behavior of the animals were similar for all groups in the study.

The mean body weight of the males in the 100 mg/kg group was below that of the controls on day 50, and from days 71 to 90 in the study. The difference was 6–7% lower than the control groups, but was not considered related to the test article because of a lack of a dose response. The mean body weight of the males in the 1000 mg/kg group was lower than the control groups, in this case, from days 22–90. No statistically significant differences in body weight were noted in the male or female rats in the 300 mg/kg group, when compared to the control. Similar effects were not observed in the female rats in any of the treatment groups. A summary of total body weights and body weight gains can be found in Table 1.

Average daily food consumption was similar in all groups except for a slightly lower mean value (*p* < 0.05) with the males in the highest dose group during week 8, and a slightly higher mean value (*p* < 0.01) with the females in the highest dose group during week 6. Water consumption for all of the male dose groups was similar when compared with the controls. Statistically significant lower water consumption (*p* < 0.05) was noted in the females in the 100 mg/kg group during days 57–58 and 88–89, and in the females in the 300 mg/kg group (*p* < 0.05) on days 57–58, 85–86 and 88–89. Females in the 1000 mg/kg dose group also had decreased water consumption, but only during days 57–58 (*p* < 0.01) and 88–89 (*p* < 0.05).

Some statistically significant differences were observed from the results of the hematology and clinical chemistry parameters tested. However, since they fell into the historically normal range for the laboratory it was concluded that no clinically relevant test item related hematological or clinical chemistry changes were observed in either the male or female rats receiving GanedenBC^30™^ at 100, 300, or 1000 mg/kg/day (Tables 2–4).

Gross pathological evaluation at the end of the study revealed several cases of pinprick-sized hemorrhages and pale, pillow-like raised areas in the lungs in all groups, which were likely caused by exsanguination of the animals. No treatment-related histopathological findings were noted upon examination of the animals at the end of the study.

Absolute organ weights differed only in the brains of the males in the 1000 mg/kg group, the liver in the 100 and 1000 mg/kg groups, and the testes in the 100 mg/kg group. However, the differences were considered to be the consequence of the lower mean body weights of these groups. Importantly, the relative organ weights compared to body weights did not differ for these organs (Table 5).

The relative kidney weight was lower than the control in the males in the 300 mg/kg group, and higher in the males in the 1000 mg/kg group (Table 5). The changes were not considered to be of biological significance or related to the test article, most importantly because they were not corroborated with any histological findings. The relative weight of the adrenal glands was lower than the control group for the females in the 300 mg/kg group, but was due to individual variation and not considered related to administration of the test article because of a lack of a dose response.

In the 90-day subchronic oral toxicity study, no toxicologically significant differences between the treated groups (100, 300 and 1000 mg/kg bw/day) and the controls were observed with respect to food consumption, water consumption, sensory reactivity, general and behavioral conditions, hematological and clinical chemistry evaluations. GanedenBC^30™^ caused neither treatment-related macroscopic or microscopic signs nor changes in the organ weights of the male and female rats at 100, 300 and 1000 mg/kg/day after the 13-week treatment period. The test item was well tolerated.

Since there were no signs of toxicity noted with respect to gross or histopathological examinations, nor with hematology, clinical chemistry, or organ weights for the 1000 mg/kg dose group, the differences in the mean body weight of the males described above is not considered related to the test article, but rather a result of biological variation. Hence the NOAEL for both males and females is considered to be >1000 mg/kg body weight per day, which was the highest dose tested.

### 3.6 Acute eye irritation study in rabbits

Ganeden BC^30™^ cell mass applied to the mucosa of the eyes resulted in slight to moderate conjunctival irritant effect within 1 h that was fully reversible in 72 h. There were no negative signs observed in either the cornea or the iris. According to EC criteria for classification and labeling requirements for dangerous substances and preparations, the test article is not required to be classified, nor is it obligatory to label the test article with regard to eye irritation.

### 3.7 Acute skin irritation study in rabbits

GanedenBC^30™^ cell mass applied to the skin resulted in very slight erythema, but no edema at 1 h after removal of the patch. At 24 h, the animals no longer had any signs of erythema. According to EC directive 2001/59/EEC the test article is not classified as irritating to the skin. The observed clinical sign of very slight erythema on the treated skin surface was concluded as fully reversible.

## 4 Discussion

New species and strains of probiotic bacteria are becoming commercially available as dietary ingredients in both dietary supplements and functional foods. It is especially important that novel probiotics are properly tested for safety and efficacy. The focus of this paper is to present a comprehensive toxicologic assessment to support the safety for chronic consumption of the novel probiotic GanedenBC^30™^. An acute oral toxicity and a 13-week subchronic oral toxicity study were conducted in Wistar rats. In addition, *in vitro* studies were conducted to evaluate mutagenicity, genotoxicity, and clastogenicity. There was no evidence for any mutagenic or genotoxic effect of GanedenBC^30™^ in either the AMES assay or the *in vitro* chromosomal aberration study. Negative results were also concluded for the micronucleus assay in mice, indicating that under the test conditions GanedenBC^30™^ does not produce cytogenetic damage. Furthermore, GanedenBC^30™^ did not produce any biologically significant skin or eye irritation.

## 5 Conclusion

In conclusion, the studies described in this paper were conducted as a comprehensive safety assessment of GanedenBC^30™^, a commercially available probiotic strain of *B. coagulans*. As part of a pre-clinical safety evaluation program, several tests have been performed. GanedenBC^30™^ demonstrated no evidence to suggest mutagenicity or genotoxicity in a number of commonly utilized genetic toxicity assays. No treatment-related mortality, morbidity or clinical symptoms resulted from an acute oral toxicity study using a single dose of 5000 mg/kg. In a subchronic oral toxicity study, GanedenBC^30™^ in daily doses of 100, 300 and 1000 mg/kg bw/day for 90 days was well tolerated and did not cause either lethality or toxic clinical symptoms in either male or female rats. The NOAEL derived from the results of the 90-day study is 1000 mg/kg. Since the concentration of the *B. coagulans* used was 1.36 × 10^11^ CFUs/g, this corresponds to 1.36 × 10^11^ CFUs/kg. For an average 70 kg human being, this corresponds to 95.2 × 10^11^ CFUs. Because the suggested human dose is in the range of 100 × 10^6^ to 3 × 10^9^ CFUs, this gives a safety factor ranging from 3173 to 95,200 times. Based upon scientific procedures and supported by history of use, GanedenBC^30™^ is considered safe for chronic human consumption.

## Conflict of interest statement

The authors declare that there are no conflicts of interest.

## Figures and Tables

**Table 1:** Summary of body weight (BW) and weekly body weight grain (BWG) data in the rat subchronic 13-week oral toxicity study.

		1	8	15	22	29	36	43	50
*Females*									
Control	BW	171.8 ± 5.37	200.6 ± 8.9	219.7 ± 13.34	238.8 ± 15.96	247.2 ± 23.64	257.5 ± 23.03	264.2 ± 20.41	278.1 ± 24.18
	BWG		28.8 ± 6.78	19.10 ± 6.54	14.10 ± 7.99	13.40 ± 11.96	10.30 ± 7.41	6.70 ± 7.30	13.90 ± 7.62
100 mg/kg/day	BW	172.8 ± 7.77	203.1 ± 7.55	232.2 ± 11.99	234.80 ± 20.95	251.5 ± 21.88	259.1 ± 18.76	271.3 ± 21.26	282.7 ± 26.17
	BWG		30.3 ± 8.31	20.10 ± 6.97	11.60 ± 11.57	16.70 ± 9.93	7.60 ± 8.13	12.20 ± 5.45	11.40 ± 8.04
300 mg/kg/day	BW	173.2 ± 7.67	205.0 ± 7.96	222.80 ± 5.49	235.6 ± 9.00	252.3 ± 11.2	263.3 ± 10.24	271.3 ± 11.87	281.4 ± 15.78
	BWG		31.8 ± 3.71	17.80 ± 10.41	12.80 ± 9.93	16.70 ± 9.32	11.00 ± 6.43	8.00 ± 6.41	10.10 ± 8.91
1000 mg/kg/day	BW	172.5 ± 5.40	200.7 ± 10.19	222.50 ± 11.10	238.5 ± 12.15	252.3 ± 17.08	262.3 ± 18.41	273.1 ± 20.06	282.4 ± 20.46
	BWG		28.2 ± 7.48	21.80 ± 8.99	16.00 ± 7.27	13.80 ± 9.43	10.00 ± 6.46	10.80 ± 7.51	9.30 ± 9.44

		57	64	71	78	85	90/91	Sum (1–91)	
Control	BW	283.2 ± 27.87	285 ± 26.03	289.2 ± 24.23	296.6 ± 27.02	297.7 ± 30.36	300.5 ± 28.04		
	BWG	5.10 ± 6.38	1.80 ± 7.50	4.20 ± 8.15	7.40 ± 6.06	1.10 ± 7.03	2.80 ± 5.96	128.70 ± 27.01	
100 mg/kg/day	BW	287.1 ± 25.92	288 ± 22.2	296 ± 26.09	302.1 ± 29.5	304.1 ± 30.78	307.5 ± 29.26		
	BWG	4.40 ± 8.45	0.90 ± 6.74	8.00 ± 6.60	6.10 ± 4.86	2.00 ± 7.85	3.40 ± 7.07	134.70 ± 28.88	
300vmg/kg/day	BW	286.4 ± 18.55	292.3 ± 15.08	295.4 ± 14.72	301.1 ± 16.15	301.7 ± 13.94	306.4 ± 14.71		
	BWG	5.00 ± 9.02	5.90 ± 7.05	3.10 ± 4.77	5.70 ± 5.12	0.60 ± 6.22	4.70 ± 6.27	133.20 ± 19.36	
1000 mg/kg/day	BW	288.7 ± 23.76	293.9 ± 21.66	301.9 ± 22.09	306.8 ± 21.80	306.7 ± 26.57	313.2 ± 23.50		
	BWG	6.30 ± 7.23	5.20 ± 6.09	8.00 ± 4.69	4.90 ± 6.24	−0.10 ± 7.87	6.50 ± 8.50	140.70 ± 23.59	

*Males*		1	8	15	22	29	36	43	50
Control	BW	210.8 ± 6.94	275.1 ± 11.18	329.3 ± 17.93	362.8 ± 21.53	396.7 ± 26.16	424.5 ± 27.00	447.1 ± 31.08	473.2 ± 34.83
	BWG		64.30 ± 6.00	54.20 ± 9.35	33.50 ± 8.64	33.90 ± 5.97	27.80 ± 5.09	22.60 ± 4.97	26.10 ± 5.07
100 mg/kg/day	BW	211.3 ± 6.02	272.2 ± 12.22	320.6 ± 18.47	354.3 ± 21.38	381.2 ± 24.22	404.3 ± 26.59	424.5 ± 33.60	445.1 ± 35.29⁎
	BWG		60.90 ± 7.02	48.40 ± 8.10	33.70 ± 4.69	26.90 ± 5.51⁎⁎	23.10 ± 3.41⁎	20.20 ± 8.13	20.60 ± 3.31⁎
300 mg/kg/day	BW	211.9 ± 5.32	272.1 ± 6.87	325.4 ± 11.15	362.5 ± 15.79	388.3 ± 17.36	417.9 ± 20.73	439.3 ± 22.21	464.1 ± 24.01
	BWG		60.20 ± 3.79	53.30 ± 6.57	37.10 ± 6.28	25.80 ± 5.45⁎⁎	29.60 ± 5.27	21.40 ± 3.72	24.80 ± 3.55
1000 mg/kg/day	BW	210.0 ± 6.55	265.6 ± 10.23	312.6 ± 15.43	344.0 ± 10.32⁎	369.5 ± 10.56⁎⁎	393.0 ± 10.17⁎⁎	411.8 ± 11.29⁎⁎	431.6 ± 13.83⁎⁎
	BWG		55.60 ± 7.17⁎⁎	47.00 ± 7.13	31.40 ± 8.14	25.50 ± 3.81⁎⁎	23.50 ± 3.72⁎	18.80 ± 5.16	19.80 ± 5.81⁎⁎

		57	64	71	78	85	90/91	Sum (1–91)	
Control	BW	482.2 ± 37.05	488.5 ± 36.56	511.2 ± 40.02	527.5 ± 40.76	539.9 ± 42.24	543.9 ± 42.05		
	BWG	9.00 ± 6.09	6.30 ± 4.11	22.70 ± 7.48	16.30 ± 5.38	12.40 ± 3.66	4.00 ± 4.74	333.10 ± 40.08	
100 mg/kg/day	BW	457.1 ± 38.79	463.9 ± 39.24	476.1 ± 38.96⁎	492.6 ± 45.33⁎	501.7 ± 45.45⁎	505.4 ± 42.80⁎		
	BWG	12.00 ± 4.35	6.80 ± 6.30	12.20 ± 5.71⁎⁎	16.50 ± 7.47	9.10 ± 4.95	3.70 ± 3.89	294.10 ± 39.08	
300 mg/kg/day	BW	483.2 ± 26.48	493.2 ± 26.74	508.9 ± 27.73	521.0 ± 29.59	534.3 ± 31.93	541.3 ± 35.09		
	BWG	19.10 ± 4.41⁎⁎	10.00 ± 4.24	15.70 ± 4.47⁎	12.10 ± 4.38	13.30 ± 4.55	7.00 ± 4.45	329.40 ± 31.60	
1000 mg/kg/day	BW	440.3 ± 12.01⁎’	451.4 ± 13.12⁎⁎	461.8 ± 15.51⁎⁎	475.3 ± 20.16⁎⁎	481.9 ± 19.58⁎⁎	487.7 ± 21.47⁎⁎		
	BWG	8.70 ± 2.98	11.10 ± 3.57⁎	10.40 ± 5.06⁎⁎	13.50 ± 8.15	6.60 ± 6.60⁎	5.80 ± 4.24	277.70 ± 21.50	

Values are mean ± S.D.

⁎ p < 0.05.

⁎⁎ p < 0.01.

**Table 2:** Summary of hematological data in the rat subchronic 13-week oral toxicity study.

Females	RBC (10^12^/L)	Hgb (g/dL)	Hct (%)	MCV(fL)	MCH (pg)	MCHC (g/dL)	RDW (%)
Control	7.98 ± 0.33	15.03 ± 0.55	41.55 ± 1.57	52.08 ± 1.80	18.81 ± 0.61	36.15 ± 0.65	11.32 ± 0.56
100 mg/kg/day	7.85 ± 0.31	15.02 ± 0.27	4170 ± 068	53.19 ± 2.27	19.18 ± 1.05	36.01 ± 0.75	11.64 ± 0.62
300 mg/kg/day	7.65 ± 0.49	14.53 ± 0.56⁎	40.12 ± 1.60⁎	52.54 ± 2.45	19.04 ± 0.83	36.22 ± 0.71	12.47 ± 2.20
1000 mg/kg/day	7.91 ± 0.53	15.06 ± 0.55	41.38 ± 135	52.42 ± 2.22	19.09 ± 0.88	36.39 ± 0.50	11.52 ± 0.44

Females	PLT (10^9^/L)	MPV (fL)	APTT (s)	PT (s)	Retic. (%)	WBC (1O^9^/L)	

Control	1034.90 ± 92.06	7.74 ± 0.58	18.25 ± 0.79	23.65 ± 1.00	2.22 ± 0.42	1.42 ± 0.32	
100 mg/kg/day	1017.70 ± 110.59	7.21 ± 0.38⁎	18.59 ± 0.54	24.06 ± 1.06	2.41 ± 0.43	1.83 ± 0.79	
300 mg/kg/day	1023.00 ± 213.31	7.23 ± 0.35⁎	18.39 ± 0.50	24.10 ± 1.28	2.97 ± 2.20	1.68 ± 0.53	
1000 mg/kg/day	996.90 ± 161.60	7.26 ± 0.43⁎	18.32 ± 1.05	23.79 ± 0.76	2.26 ± 0.54	2.33 ± 1.60	

Males	RBC (10^12^/L)	Hgb (g/dL)	Hct (%)	MCV (fL)	MCH (pg)	MCHC (g/dL)	RDW (%)

Control	8.69 ± 0.41	15.66 ± 0.61	43.85 ± 1.92	50.46 ± 1.11	18.02 ± 0.37	35.70 ± 0.31	12.51 ± 0.46
100 mg/kg/day	8.60 ± 0.45	15.48 ± 0.58	42.97 ± 1.62	50.01 ± 1.73	18.04 ± 0.69	36.10 ± 0.45	12.06 ± 0.48⁎
300 mg/kg/day	8.56 ± 0.36	15.74 ± 0.74	43.61 ± 2.04	50.97 ± 1.22	18.40 ± 0.48	36.08 ± 0.42	12.46 ± 0.28
1000 mg/kg/day	8.73 ± 0.40	15.53 ± 0.61	43.60 ± 1.64	49.97 ± 1.67	17.80 ± 0.61	35.62 ± 0.58	12.53 ± 0.55

Males	PLT (10^9^/L)	MPV (fL)	APTT (s)	PT (s)	Retic. (%)	WBC (1O^9^/L)	

Control	1030.90 ± 136.70	7.69 ± 0.91	17.65 ± 0.62	25.56 ± 0.89	1.93 ± 0.16	3.40 ± 0.95	
100 mg/kg/day	957.20 ± 72.51	7.70 ±0.82	17.61 ± 0.79	25.20 ± 0.89	1.87 ± 0.30	4.01±1.47	
300 mg/kg/day	989.00 ± 106.87	7.70 ± 0.48	18.19 ± 0.75	25.48 ± 1.23	2.21 ± 0.21⁎	3.20 ± 1.00	
1000 mg/kg/day	1104.30 ± 161.76	7.77 ± 0.61	17.63 ± 1.50	26.53 ± 0.89⁎	1.86 ± 0.30	3.48 ± 1.65	

Values are mean ± S.D.

⁎ *p* < 0.05.

**Table 3:** Summary of male serum biochemical data in the rat subchronic 13-week oral toxicity study.

Males	Glucose (mmol/l)	Urea (mmol/l)	Creat. (umol/l)	Na^+^ (mmol/l)	K^+^ (mmol/l)	Cl^−^ (mmol/L)	Ca^++^ (mmol/l)	P (mmol/l)	T. Chol. (mmol/l)
Control	6.94 ± 0.95	6.80 ± 0.94	54.62 ± 6.58	145.61 ± 1.01	5.28 ± 0.49	105.19 ± 1.49	2.47 ± 0.05	2.42 ± 0.25	1.46 ± 0.26
100 mg/kg/day	6.81 ± 1.22	7.67 ± 1.28	58.85 ± 9.79	145.20 ± 0.51	5.06 ± 0.45	104.66 ± 1.45	2.41 ± 0.06⁎	2.39 ± 0.18	1.58 ± 0.15
300 mg/kg/day	6.85 ± 1.44	6.98 ± 0.62	54.69 ± 4.46	144.81 ± 0.71	4.99 ± 0.46	104.7 ± 1.45	2.43 ± 0.06	2.34 ± 0.17	1.60 ± 0.36
1000 mg/kg/day	6.74 ± 0.90	6.81 ± 0.63	54.81 ± 4.13	145.19 ± 1.07	5.35 ± 0.35	104.20 ± 1.16	2.40 ± 0.08⁎	2.360 ± 0.17	1.68 ± 0.28

Males	T. Prot. (g/L)	Alb (g/L)	AST (U/L0)	ALT (U/L)	ALKP (U/L)	GGT (U/L)	T. Bil. (umol/L)	A/G	

Control	55.84 ± 1.58	29.79 ± 1.09	119.20 ± 13.74	53.00 ± 4.32	90.90 ± 13.47	6.00 ± 0.00	3.49 ± 0.21	1.16 ± 0.07	
100 mg/kg/day	54.69 ± 2.12	28.68 ± 2.09	128.40 ± 19.40	54.60 ± 9.37	87.60 ± 12.57	6.00 ± 0.00	3.04 ± 0.36	1.10 ± 0.08⁎	
300 mg/kg/day	53.95 ± 2.02⁎	28.08 ± 1.43⁎	120.70 ± 16.67	54.80 ± 3.29	83.50 ± 11.25	6.00 ± 0.00	3.47 ± 0.63	1.08 ± 0.04⁎	
1000 mg/kg/day	55.98 ± 1.69	28.96 ± 0.97	134.70 ± 32.44	55.80 ± 4.18	90.50 ± 16.57	6.00 ± 0.00	3.32 ± 0.72	1.08 ± 0.06⁎	

Values are mean ± S.D. ^∗∗^*p* < 0.01.

⁎ *p* < 0.05.

**Table 4:** Summary of female serum biochemical data in the rat subchronic 13-week oral toxicity study.

Females	Glucose (mmol/l)	Urea (mmol/l)	Creat. (umol/l)	Na^+^ (mmol/l)	K^+^(mmol/l)	Cl^−^ (mmol/l)	Ca^++^(mmol/l)	P (mmol/l)	T. Chol. (mmol/l)
Control	6.55 ± 0.86	6.87 ± 0.80	57.83 ± 7.35	144.78 ± 1.29	4.64 ± 0.38	105.05 ± 1.69	2.49 ± 0.10	1.96 ± 0.24	1.83 ± 0.37
100 mg/kg/day	7.44 ± 0.89	7.21 ± 1.73	56.13 ± 7.49	143.79 ± 1.49	4.40 ± 0.44	103.65 ± 2.60	2.43 ± 0.08	1.74 ± 0.39	1.69 ± 0.34
300 mg/kg/day	6.72 ± 0.52	7.06 ± 1.34	53.95 ± 4.10	144.58 ± 1.74	4.40 ± 0.32	105.55 ± 1.33	2.43 ± 0.06	1.85 ± 0.33	1.49 ± 0.26
1000 mg/kg/day	7.06 ± 0.69	7.58 ± 1.16	57.77 ± 7.27	144.85 ± 1.42	4.44 ± 0.37	105.70 ± 1.96	2.43 ± 0.07	2.03 ± 0.25	1.77 ± 0.47

Females	T. Prot. (g/l)	Alb (g/l)	AST (U/L0)	ALT (U/L)	ALKP (U/L)	GGT (U/L)	T. Bil. (Mmol/l)	A/G	
Control	54.63 ± 3.50	31.06 ± 3.07	115.50 ± 19.28	46.90 ± 5.82	55.80 ± 11.04	6.00 ± 0.00	4.05 ± 0.66	1.34 ± 0.15	
100 mg/kg/day	54.78 ± 2.81	31.02 ± 1.92	120.60 ± 46.93	50.20 ± 11.35	52.80 ± 5.07	6.00 ± 0.00	3.80 ± 0.37	1.31 ± 0.12	
300 mg/kg/day	53.60 ± 2.11	28.97 ± 1.95	100.80 ± 17.05	49.80 ± 4.71	56.30 ± 10.56	6.00 ± 0.00	3.55 ± 0.46	1.19 ± 0.10⁎⁎	
1000 mg/kg/day	52.80 ± 1.60	28.97 ± 1.23⁎	111.30 ± 22.19	45.50 ± 4.40	57.20 ± 9.04	6.00 ± 0.00	3.47 ± 0.62⁎	1.22 ± 0.06⁎	

Values are mean ± S.D.

⁎ *p* < 0.05.

⁎⁎ *p* < 0.01.

**Table 5:** Summary of mean organ weights (g) and organ weight relative to body weight (%) in the rat subchronic 13-week oral toxicity study.

		Control	100 mg/kg/day	300 mg/kg/day	1000 mg/kg/day
*Males*					
Body weight	g	522.20 ± 38.94	497.00 ± 66.06	523.70 ± 30.98	470.20 ± 20.87⁎⁎
Brain (g)	g	2.23 ± 0.08	2.23 ± 0.14	2.20 ± 0.06	2.14 ± 0.06⁎
	%	0.430 ± 0.036	0.452 ± 0.048	0.422 ± 0.030	0.456 ± 0.024
Liver	g	12.40 ± 1.44	11.29 ± 1.23⁎	12.35 ± 0.93	11.19 ± 0.78⁎
	%	2.371 ± 0.146	2.283 ± 0.193	2.358 ± 0.106	2.379 ± 0.110
Heart	g	1.38 ± 0.16	1.27 ± 0.11	1.35 ± 0.06	1.29 ± 0.09
	%	0.265 ± 0.021	0.258 ± 0.027	0.257 ± 0.013	0.275 ± 0.017
Spleen	g	1.13 ± 0.21	0.97 ± 0.18	1.04 ± 0.11	1.01 ± 0.14
	%	0.216 ± 0.034	0.196 ± 0.024	0.199 ± 0.020	0.215 ± 0.026
Kidneys	g	3.02 ± 0.34	2.81 ± 0.17	2.82 ± 0.20	2.93 ± 0.20
	%	0.576 ± 0.029	0.569 ± 0.051	0.538 ± 0.035⁎	0.623 ± 0.038⁎
Thymus	g	0.45 ± 0.08	0.35 ± 0.11	0.41 ± 0.49	0.36 ± 0.12
	%	0.086 ± 0.014	0.070 ± 0.017	0.078 ± 0.018	0.075 ± 0.024
Testes	g	4.10 ± 0.48	3.68 ± 0.32⁎	3.76 ± 0.21	3.78 ± 0.36
	%	0.786 ± 0.082	0.748 ± 0.086	0.720 ± 0.051	0.805 ± 0.070
Epididymides	g	1.57 ± 0.15	1.46 ± 0.11	1.50 ± 0.18	1.59 ± 0.24
	%	0.301 ± 0.033	0.297 ± 0.038	0.288 ± 0.041	0.337 ± 0.047
Adrenals	g	0.065 ± 0.009	0.061 ± 0.008	0.065 ± 0.011	0.064 ± 0.006
	%	0.0126 ± 0.0021	0.0124 ± 0.0019	0.0125 ± 0.0023	0.0137 ± 0.0013

*Females*					
Body weight	g	284.70 ± 27.35	294.40 ± 28.78	292.60 ± 16.02	296.40 ± 22.97
Brain	g	2.01 ± 0.06	2.03 ± 0.10	1.99 ± 0.09	2.03 ± 0.08
	%	0.711 ± 0.065	0.695 ± 0.065	0.681 ± 0.039	0.688 ± 0.055
Liver	g	6.84 ± 0.91	7.52 ± 0.85	7.36 ± 1.35	7.24 ± 0.73
	%	2.394 ± 0.118	2.556 ± .0209	2.506 ± 0.379	2.441 ± 0.145
Heart	g	0.87 ± 0.08	0.91 ± 0.14	0.88 ± 0.09	0.89 ± 0.06
	%	0.308 ± 0.024	0.310 ± 0.042	0.303 ± 0.032	0.299 ± 0.019
Spleen	g	0.67 ± 0.08	0.75 ± 0.14	0.77 ± 0.17	0.73 ± 0.10
	%	0.236 ± 0.027	0.256 ± 0.048	0.260 ± 0.050	0.246 ± 0.029
Kidneys	g	1.77 ± 0.18	1.85 ± 0.17	1.81 ± 0.16	1.90 ± 0.18
	%	0.623 ± 0.038	0.634 ± 0.077	0.617 ± 0.045	0.639 ± 0.035
Thymus	g	0.27 ± 0.05	0.31 ± 0.06	0.27 ± 0.07	0.32 ± 0.07
	%	0.096 ± 0.018	0.104 ± 0.022	0.092 ± 0.024	0.108 ± 0.021
Uterus	g	0.80 ± 0.23	0.85 ± 0.23	0.78 ± 0.26	0.91 ± 0.34
	%	0.287 ± 0.097	0.294 ± 0.106	0.269 ± 0.094	0.312 ± 0.124
Ovaries	g	0.144 ± 0.030	0.147 ± 0.010	0.147 ± 0.015	0.142 ± 0.030
	%	0.0509 ± 0.0113	0.0505 ± 0.0074	0.0504 ± 0.0065	0.0480 ± 0.0095
Adrenals	g	0.094 ± 0.011	0.094 ± 0.012	0.085 ± 0.011	0.091 ± 0.012
	%	0.0333 ± 0.0041	0.0321 ± 0.0031	0.0289 ± 0.0034⁎	0.0307 ± 0.0039

Values are mean ± S.D.

⁎ *p* < 0.05.

⁎⁎ *p* < 0.01.
